# High Rates of Multimorbidity Reported Among People of Color Despite Healthy Weight

**DOI:** 10.1089/heq.2022.0074

**Published:** 2022-09-01

**Authors:** Carlos T. Jackson, Jessica Young, W. Moraa Onsando

**Affiliations:** ^1^ Community Care of North Carolina, Cary, North Carolina, USA.; ^2^ The Dartmouth Institute of Health Policy and Clinical Practice, Dartmouth College, Hanover, New Hampshire, USA.; ^3^ Department of General Pediatrics and Adolescent Medicine, Department of Family Medicine, and Preventive Medicine Residency Program, University of North Carolina at Chapel Hill, Chapel Hill, North Carolina, USA.

**Keywords:** multimorbidity, racial disparities, body mass index, BRFSS survey

## Abstract

**Purpose:**

Weight management is one of the most cited levers for preventing and managing many chronic conditions, particularly those considered to be “lifestyle modifiable.” However, it is unclear how much weight is a driver of illness burden among people of color. This article sought to examine whether people of color are more likely to develop “lifestyle-modifiable” conditions, including diabetes, kidney disease, heart disease, lung disease, and hypertension, both individually and in combination (multimorbidity), in the absence of being obese.

**Methods:**

Using data from the 2019 Behavioral Risk Factors Surveillance System survey, we examined the risk of having these conditions among Black, Asian, Native American, Latino/a, and White respondents who reported being “normal weight” (*n*=86,682), while also controlling for age, gender, smoking history, physical activity, and diet.

**Results:**

For each individual condition, White respondents almost always had the lowest risk. On the other hand, Latino/a respondents had the highest rates of diabetes and kidney disease. Native American respondents had the highest rates of heart and lung disease. Black respondents had the highest rates of hypertension. Despite an otherwise healthy weight, Native American, Black, and Latino/a people were 2.5, 2.3, and 1.8 times, respectively, more likely to develop multiple chronic conditions that are typically considered “lifestyle modifiable,” compared to White people.

**Conclusion:**

Disease prevention and management guidelines driven by the clinical experience of White people are insufficient for addressing the considerable illness burden that people of color continue to experience.

## Introduction

Multimorbidity—having two or more chronic conditions at the same time—is a growing concern and associated with some of the worst outcomes compared to any one individual condition.^1^ For any given condition, having a second condition at the same time results in a substantially more challenging treatment regimen and poorer outcomes overall. Indeed, during the recent covid pandemic, having multiple chronic conditions placed individuals at much greater risk of infection.^2^

It is well documented that chronic conditions disproportionately affect people of color.^3,4^ For example, people of color have long been at higher risk of developing diabetes, and the disparity between these populations and White individuals has only increased in recent decades.^5^ Disparities in disease management have been identified in various care settings and across several conditions,^6–9^ persisting even when controlling for income, education, insurance status, patient preference, and access to care, and this increased predisposition is confounded by several social determinants of health.^10,11^ Racial and ethnic minority patients are less likely to receive cardiac catheterization for cardiovascular disease, necessary kidney transplants, adequate pain management, and appropriate mental health services or cancer screening, to name a few.^12^

These patients are at increased risk both for developing chronic diseases and mortality compared to their white counterparts.^4,13,14^ Although research into the understanding of multimorbidity among different racial and ethnic groups in America is limited, two studies found Black patients to have a greater risk of multimorbidity compared to other groups.^15,16^ However, these studies notably did not stratify by body mass index (BMI), and considered more conditions than just those typically considered “lifestyle modifiable.” Not taking into account lifestyle modifications like weight, diet, and exercise, results from such studies can lead to erroneous conclusions that more lifestyle management is necessary to address these disparities.^17^ Also, in this study, the term people of color is used broadly to describe a group of people who identify as Black, Asian, Native American, and/or Latino/a.

Although people of color is used in this context to highlight health disparities across different races and ethnicities, we acknowledge the challenges with using this term to describe a diverse group of individuals, including some who may not identify as a person of color.

Multimorbidity is linked to obesity, particularly for conditions that are considered lifestyle modifiable,^18–20^ although this relationship has been less well studied, and rarely in a population of normal-weight individuals. To date, BMI has been demonstrated to be one of the strongest predictors of developing diabetes, perhaps even greater than genetics.^21^ Likewise, the National Kidney Foundation report a strong association between weight and kidney disease,^22^ and results from a very large study of over 1 million adults living in United Kingdom found a direct link between increased BMI and risk of chronic kidney disease, independent of other risk factors or chronic conditions.^23^

The American Heart Association also reports a strong association between obesity and cardiovascular risk factors, as well as direct links between weight and cardiovascular disease and mortality.^24^ Although often underappreciated, there are demonstrated links between obesity and the development and severity of lung disease, including asthma and chronic obstructive pulmonary disease (COPD).^25^

Weight status is often cited as a major risk factor for chronic diseases, with clinical recommendations typically prioritizing overweight and obese individuals for screening and early disease detection, allowing for early intervention and ultimately prevention of further morbidity and mortality. Indeed, weight loss is considered one of the top, if not the top, behavioral changes a person can make to prevent and manage diabetes,^26^ kidney disease,^27^ heart disease,^28^ and hypertension.^29^ Although to a lesser extent, weight loss has also been recommended to prevent and manage lung disease.^30^

While it is true that weight loss can help, focusing on this one lifestyle modification potentially minimizes the powerful impact of other forces at play among people of color. Coupled with findings that people of color are more likely to be obese^31^ may inadvertently mask the unique effects of race and ethnicity on the development of chronic health conditions. However, recent literature has questioned whether the decision to screen for diseases based on BMI alone can effectively prevent morbidity and mortality from chronic disease development for all populations, especially for people of color.

Despite the significant role that BMI plays in terms of disease risk, Zhu et al^32^ recently reported findings from their study that showed people of color are at increased risk of diabetes even at a lower BMI. Subjects in their study were clustered within a handful of health systems across the country, and they did not control for smoking, diet, and exercise, they only looked at diabetes and pre-diabetes. However, their conclusions have profound implications as far as the prevention and management of chronic health conditions that have long been believed to be driven by lifestyle, and suggest that there may be further need to investigate other risk factors that are equally important as weight status and independent of lifestyle behaviors.

The purpose of this study is to evaluate the risk of multiple chronic health conditions among Black, Asian, Native American, Latino/a, and White persons, but specifically among that subset who reported being at a healthy weight. Given that people of color have a heightened risk of diabetes at a normal weight, it is reasonable to question whether the same might be true for other lifestyle-modifiable diseases and ultimately for the development of multiple comorbid health conditions.

## Methods

### Data

Data were obtained from the 2019 version of the Behavioral Risk Factors Surveillance System (BRFSS) survey. Sponsored by the Center for Disease Control and Prevention (CDC) since 1984, the BRFSS is a collaboration of individual states and the federal government to monitor the presence of a multitude of behavioral risk factors and chronic health conditions. Based on a probabilistic sampling strategy, BRFSS annually employs random-digit-dialing of landlines and cell phone numbers to identify a nationally representative sample of English and Spanish speakers.^33^ Since 2011, BRFSS has used a weighting methodology known as iterative proportional fitting or raking. Raking has several advantages, including the allowance for more demographic variables, including the variable of telephone ownership (landline or cellular telephone), in the statistical weighting process. This further reduces the potential for biased estimates.

### Outcome variable

Our primary outcome measures were the self-report of the presence of five chronic conditions, as well as the likelihood of reporting that an individual has two or more of these conditions (multimorbidity). All outcomes were converted to a binary variable. Diabetes was based on self-report responses to the question, “Has a doctor, nurse, or other health professional ever told you that you had diabetes?” Responses that were coded “Yes” were used to indicate the presence of diabetes.

Responses that indicated either pre-diabetes or gestational diabetes only were recoded to “No.” Hypertension was based on self-report responses to the question, “Have you ever been told by a doctor, nurse, or other health professional that you have high blood pressure?” Kidney disease was based on self-report responses to the question, “Not including kidney stones, bladder infection or incontinence, were you ever told you had kidney disease?” Heart disease was based on self-report responses to the question, “(Ever told) you had angina or coronary heart disease?” or “(Ever told) you had a heart attack, also called a myocardial infarction?”; a positive response to either question indicated heart disease.

Lung disease was based on self-report responses to the question, “(Ever told) (you had) chronic obstructive pulmonary disease, C.O.P.D., emphysema, or chronic bronchitis?” or a positive response to both of the following questions, “(Ever told) you had asthma?” and “Do you still have asthma?” Lung disease was indicated if the respondent acknowledged having either COPD or asthma and was treated as a single condition in the analyses. Although a respondent could potentially have more than one diagnosis that can map to the same broad condition categories, for the purposes of these analyses, conditions were counted based on the presence or absence of each of the broad categories of conditions. Mutimorbidity was defined as having two or more of the condition categories that were assessed.

### Sample

Respondents were included in the analyses if they were between 18 and 74 years of age, reported a height and weight that placed them in the normal range for BMI (18.5–24.9), and reported that they were either Asian, Black, Latino/a, Native American, or White. Respondents who reported being a different race or mixed race were not included in these analyses. All individuals reporting that they were “Hispanic” were included in the Latino/a group, regardless of which race they indicated. Respondents were also excluded if they were missing data for any of the predictor or outcome variables used in the logistic regression models. Because of the high correlation between morbidity and mortality, particularly after age 74, and the substantial differences in mortality rates across racial/ethnic groups, we excluded respondents 75 years of age and older. BMI was calculated based on self-reported weight and height.

### Predictor variables

For the age variable, we converted the age categories to the median value for the category so that we could treat it as a continuous variable (e.g., respondents 30–34 years of age were treated as 32.5 years old). Gender was based on responses to the question, “Are you male or female?” Smoking history was based on responses to the question, “Have you smoked at least 100 cigarettes in your entire life?” with a response of “No” being considered “Never Smoked.” Physically active respondents were identified by the calculated categories of “Highly Active” or “Active.” Healthy eating was defined as consuming an average of five or more fruits or vegetables per day.

## Analysis

We conducted a series of logistic regression analyses that estimated the likelihood of a person reporting that they had diabetes, kidney disease, heart disease, lung disease, hypertension, or multimorbidity. Each model also controlled for age, gender, smoking history, physical activity, and healthy eating. In addition, our models employed the statistical weights included with the BRFSS public-use dataset to maximize the representativeness of the sample to the general population of normal-weight Americans. All analyses were conducted using SAS version 9.4 (Cary, NC). BRFSS survey data are considered public-use data that are not human subjects research and do not require approval from an Institutional Review Board.

## Results

Data from 86,682 survey respondents were included in the analysis. The racial/ethnic breakdown was as follows: 3,588 Asian, 5,271 Black, 7,740 Latino/a, 1,187 Native American, and 68,896 White. There were slightly more females in every group, and the White and Native American respondents tended to be older than the Black, Asian, and Latino/a respondents. Asian and Latino/a respondents were much more likely to never have smoked, compared to Black, White, and Native American respondents, which may have been due to those groups skewing younger than the others.

White and Native American respondents tended to be more likely to report that they were physically active compared to Asian, Latino/a, and Black respondents. Relatively few respondents reported eating at least five fruits or vegetables each day, ranging between 15% and 17% across each of the groups. See Table 1 for more details, including the unadjusted rates for each of the outcomes being studied.

**Table 1.** Sample Characteristics

Variable	Asian	Black	Latino/a	Native American	White	Grand total
*n*	3,588	5,271	7,740	1,187	68,896	86,682
Median age	37.5	47.5	37.5	52.5	52.5	52.5
% Female	1,861 (51.9%)	2712 (51.5%)	4,332 (56.0%)	652 (54.9%)	41,853 (60.7%)	51,410
% Never smoked	2,799 (78.0%)	3,148 (59.7%)	5,462 (70.6%)	473 (39.8%)	39,602 (57.5%)	51,484
% Physically active	1,679 (46.8%)	2,159 (41.0%)	3,347 (43.2%)	598 (50.4%)	39,324 (57.1%)	47,107
% Healthy eating	558 (15.6%)	908 (17.2%)	1,349 (17.4%)	200 (16.8%)	10,601 (15.4%)	13,616
Unweighted rates						
% Diabetes	201 (5.6%)	518 (9.8%)	532 (6.9%)	108 (9.1%)	2,772 (4.0%)	4,131
% Heart disease	84 (2.3%)	295 (5.6%)	274 (3.5%)	112 (9.4%)	3,074 (4.5%)	3,839
% Hypertension	589 (16.4%)	1,770 (33.6%)	1,285 (16.6%)	336 (28.3%)	14,499 (21.0%)	18,479
% Kidney disease	51 (1.4%)	190 (3.6%)	173 (2.2%)	50 (4.2%)	1,403 (2.0%)	1,867
% Lung disease	178 (4.9%)	743 (14.1%)	728 (9.4%)	244 (20.6%)	8,501 (12.3%)	10,391
% Multimorbidity	203 (5.7%)	871 (16.5%)	620 (8.0%)	219 (18.4%)	6,045 (8.8%)	7,958

Table 2 presents the odds ratios from the logistic regression models. For every model, older age, being male, having smoked, and not being physically active were associated with a greater likelihood of that condition. Paradoxically, healthy eating was associated with a higher risk, although only hypertension was statistically significant.

**Table 2.** Odds Ratios from Logistic Regression Models

Outcome	Diabetes		Heart disease		Hypertension		Kidney disease		Lung disease		Multimorbidity	
Age	1.07	^a^	1.07	^a^	1.06	^a^	1.05	^a^	1.01	^a^	1.07	^a^
Gender (male)	1.38	^a^	1.97	^a^	1.49	^a^	1.21	^b^	0.78	^a^	1.40	^a^
Never smoked	0.74	^a^	0.45	^a^	0.61	^a^	0.65	^a^	0.47	^a^	0.43	^a^
Physically active	0.58	^a^	0.65	^a^	0.81	^a^	0.70	^a^	0.66	^a^	0.56	^a^
Healthy eating	1.08	n.s.	1.05	n.s.	1.16	^b^	0.98	n.s.	1.09	n.s.	1.07	n.s.
Asian	1.92	^a^	1.21	n.s.	0.91	n.s.	0.99	n.s.	0.48	^a^	1.13	n.s.
Black	2.75	^a^	1.22	n.s.	2.32	^a^	1.61	^b^	1.27	^a^	2.31	^a^
Latino/a	3.50	^a^	1.43	^b^	1.37	^a^	1.83	^a^	0.86	^b^	1.81	^a^
Native American	2.00	^b^	2.41	^a^	1.43	^b^	1.74	n.s.	1.76	^a^	2.50	^a^
Model c-statistic	0.766		0.793		0.755		0.695		0.652		0.793	

^a^ *p*<0.001.

^b^ *p*<0.05.

n.s., not significant.

Among a sample of reportedly healthy individuals, logistic regression models found that Black people had a higher risk of diabetes, kidney disease, lung disease, and hypertension; Latino/a people had a higher risk of diabetes, kidney disease, heart disease, and hypertension; Native American people had a higher risk of diabetes, heart disease, lung disease, and hypertension; and Asian people only had a higher risk of diabetes. Black, Latino/a and Native American respondents were 2.3, 1.8, and 2.5 times more likely to have multimorbidity compared to White respondents, respectively.

Table 3 displays the adjusted rates for each chronic condition and multimorbidity by racial/ethnic group. The rates reported reflect what would be predicted for an average 60-year-old normal-weight respondent as a function of their race/ethnicity; rates control for average gender, smoking history, physical activity, and diet. For nearly every condition, White respondents have the lowest probability of having that condition, and in most cases, their risk is substantially lower than that observed among other racial/ethnic groups. Latino/a respondents have the highest rates of diabetes and kidney disease. Native American respondents have the highest rates of heart disease and lung disease. Black respondents have the highest rates of hypertension.

**Table 3.** Adjusted Disease Rates for an Average 60-Year-Old Person with Normal Body Mass Index

	Diabetes	Heart disease	Hypertension	Kidney disease	Lung disease	Multimorbidity
Asian	10%	7%	33%	3%	7%	13%
Black	14%	7%	85%	4%	18%	27%
Latino/a	18%	8%	50%	5%	12%	21%
Native American	10%	14%	52%	5%	25%	29%
White	5%	6%	37%	3%	14%	12%

## Discussion

We believe this is among the first article of its kind to evaluate the risk of multimorbidity—defined here as having multiple lifestyle-modifiable conditions—specifically among normal-weight Americans. In addition to having a greater likelihood of any of the individual conditions evaluated, our study demonstrates that individuals with normal BMI, who identify as Native American, Black, or Latino/a, were 2.5, 2.3, and 1.8 times more likely to have multimorbidity compared to White respondents, respectively. The risks of developing diabetes, heart disease, lung disease, and hypertension were higher for respondents who were Native American, Black, or Latino/a, while Asian respondents had a higher risk of diabetes alone. This article builds on the growing body of evidence demonstrating race and ethnicity-based differences in risk of clinically serious and costly chronic health conditions, despite engaging in healthy behaviors.

Despite being in line with those lifestyle recommendations typically prescribed for people with these conditions, when compared to White members of the population, people of color experience substantially greater illness burden from conditions traditionally considered to be “lifestyle modifiable.” Clinical guidelines across the spectrum need to examine implicit biases present, which may be optimized for the average White person, but be insufficient for people of color. Most clinical guidelines have historically been based on experiences with White patients, thus minimizing the much greater risk inherent to people of color.

This article suggests that prevention and screening for chronic health conditions should take place earlier and at a lower weight threshold for people of color than what has historically been suggested for White patients. This article also supports the findings from an earlier study,^32^ but builds on that work by also controlling for smoking, diet, and physical activity and examining multiple chronic conditions.

Findings from this study and others like it would suggest the need to go beyond just the goal of health “equity” to calling for the need for a better understanding of the screening and management needs of underrepresented populations to reduce the risk of disease development and progression. Earlier and more frequent screening of common health conditions, like those evaluated in this study, would seem to be indicated for patients from racially and ethnically diverse backgrounds. Furthermore, the findings from this study underscore that there are other risk factors that serve as significant drivers of chronic disease among people of color. Like many health disparities, there are likely social determinants of health that contribute to these differences, which are not accounted for in many current clinical guidelines.^10,11^

Recent literature has examined associations between chronic stress and the development of chronic disease. One large international study observed that psychosocial stressors (stress at work and at home, financial stress, and major life events in the past year) were associated with development of heart disease when stressors were examined separately.^34^ Further studies have continued to explore plausible physiological processes that occur secondary to chronic stress and directly lead to cardiovascular disease.^35^ Furthermore, there is a body of growing research that suggests adverse childhood experiences (ACEs), experiences involving abuse and household dysfunction that occurred during one's childhood, may be linked to disease later in life.

Gilbert et al^36^ analyzed data from the 2010 BRFSS and reported that the odds of having a chronic disease was increased in those reporting at least one ACE compared to those not reporting any ACE. Sonu et al^37^ reported that four or more ACEs were associated with an increased risk of early-onset chronic diseases, and higher percentages were reported among those identifying as Black, American Indian/Alaskan native, Hispanic, and multiracial when compared to White Americans.^37^ These findings support that there are other, and often unmeasured, factors that meaningfully contribute to the development of chronic diseases, and these factors likely affect health outcomes of vulnerable populations across America.

This study found that nearly 30% of people of color suffer from multiple “lifestyle-modifiable” conditions by age 60, despite exhibiting an objectively healthy lifestyle. Greater efforts must be made to break down the entrenched barriers to care and systemic racism, which continue to exist in America. Earlier and more frequent screening of common health conditions like those evaluated in this study would seem to be indicated for all people of color.

The strengths of the study are the large, diverse, nationwide sample, which afforded us the ability to focus on a subsection of the population with a normal BMI, while controlling for other behaviors, including smoking, physical activity, and diet. The most notable limitations are the self-report nature of the study. However, data from a large, longitudinal study, the Atherosclerosis Risk in Communities study, found that self-reported diabetes diagnoses were reasonably accurate, with a 92–95% accuracy.^38^ Recent studies from United Kingdom and the United States provide evidence for the reliability of self-reported BMI from self-reported height and weight measurements^39^ and that for the vast majority of the population, BMI categories are fairly stable over time.^40^ Analyses were limited to only five race/ethnicity categories and five chronic conditions. However, future studies should also look at these patterns for other racial groups and chronic conditions.

## Health Equity Implications

Despite a reportedly healthy weight, people of color experience a burden of illness from multiple “lifestyle-modifiable” conditions that markedly exceed that of a similar White person. Clinical guidelines that were primarily developed around a majority White population are insufficient for people of color. Newer strategies and interventions that have a greater impact on the drivers of such disparity are necessary to help people of color maintain optimal health.

## Abbreviations Used

ACEs: adverse childhood experiences
BMI: body mass index
BRFSS: Behavioral Risk Factors Surveillance System
CDC: Center for Disease Control and Prevention
COPD: chronic obstructive pulmonary disease
n.s.: not significant
